# Aberrant methylation of suppressor of cytokine signalling-1 (*SOCS-1*) gene in pancreatic ductal neoplasms

**DOI:** 10.1038/sj.bjc.6601039

**Published:** 2003-07-15

**Authors:** N Fukushima, N Sato, F Sahin, G H Su, R H Hruban, M Goggins

**Affiliations:** 1 Department of Pathology, The Johns Hopkins Medical Institutions, 632 Ross Building, 720 Rutland Ave, Baltimore, MD 21205-2196, USA; 2 Department of Oncology, The Johns Hopkins Medical Institutions, 632 Ross Building, 720 Rutland Ave, Baltimore, MD 21205-2196, USA; 3 Department of Medicine, The Johns Hopkins Medical Institutions, 632 Ross Building, 720 Rutland Ave, Baltimore, MD 21205-2196, USA

**Keywords:** CpG island methylation, *SOCS-1*, JAK/STAT pathway, IL-6, pancreatic ductal adenocarcinoma

## Abstract

The suppressor of cytokine signalling-1 (*SOCS-1*) gene is frequently silenced in human hepatocellular carcinoma by aberrant methylation. The aim of this study was to determine if *SOCS-1* is inactivated in pancreatic ductal neoplasms, and to investigate if aberrant methylation of this gene affected the Janus kinase/signal transducers and activators of transcription (JAK/STAT) pathway. Aberrant methylation in the CpG island of the *SOCS-1* gene was detected in six of 19 (31.6%) human pancreatic cancer cell lines using methylation-specific PCR, and was associated with a loss or reduction of gene expression in five of the six methylated cell lines. Thirteen of 60 pancreatic ductal adenocarcinomas (21.7%) and two of 34 intraductal papillary mucinous neoplasms (IPMNs) (5.9%) had methylated *SOCS-1*. In contrast, *SOCS-1* methylation was not seen in pancreatic normal ductal epithelia (zero out of 15), in pancreatic intraepithelial neoplasia (PanINs) (zero out of 49) or in the IPMNs without infiltrating cancer (zero out of 20). 5-Aza-2′-deoxycytidine treatment of the *SOCS-1*-methylated pancreatic cancer cell lines led to restoration of *SOCS-1* gene expression. Interleukin-6, which has been shown to act through the JAK/STAT pathway to increase cell growth, induced modest time and dose-dependent cell proliferation in a *SOCS-1*-methylated cell line (PL10, *P*=0.015) but not in two unmethylated cell lines. These results indicate that loss of *SOCS-1* gene is associated with transcriptional silencing and may have growth-promoting effects, and that its methylation is a useful marker of pancreatic cancer.

Pancreatic ductal adenocarcinoma is a highly malignant neoplasm that still carries an extremely poor prognosis. Most patients are diagnosed at an advanced stage of disease, and the incidence and mortality rates for invasive pancreatic cancer are almost identical. The 5-year survival rate is 0–2% (Klöppel, 2000). Characterisation of the genetic and epigenetic changes involved in the pathogenesis of pancreatic cancer should help in the development of sensitive early detection strategies.

Suppressor of cytokine signalling-1 (SOCS-1) is a cytokine-inducible protein that functions to regulate negatively cytokine signal transduction pathways by directly interacting with Janus kinase (JAK) (Endo *et al*, 1997; Naka *et al*, 1997; Starr *et al*, 1997). The SH2 domain of SOCS-1 binds to a JH1 domain of JAK2 and inhibits the phosphorylation of JAK2, thus downregulating the JAK/ signal transducers and activators of transcription (STAT) pathway (Narazaki *et al*, 1998; Yasukawa *et al*, 1999). Several studies have shown that constitutive overexpression of SOCS-1 inhibits signalling in response to a range of cytokines, including interferon-*γ* (Brysha *et al*, 2001), tumour necrosis factor-*α* (Morita *et al*, 2000), interleukin-2 (IL-2) (Sporri *et al*, 2001), IL-4 and IL-6 (Starr *et al*, 1997).

Interleukin-6 has been implicated as a mediator of growth control in several human neoplasms including multiple myeloma (Hussein *et al*, 2002), prostatic cancer (Giri *et al*, 2001) and colonic cancer (Schneider *et al*, 2000) and elevation of serum IL-6 level have been reported in some cancers such as renal cell carcinoma (Blay *et al*, 1992), ovarian cancer (Plante *et al*, 1994), cholangiocarcinoma (Goydos *et al*, 1998) and pancreatic cancer (Barber *et al*, 1999). The binding of IL-6 to its receptor induces homodimerisation of gp130, which leads to cross-phosphorylation and activation of the associated JAK2. Signal transducers and activators of transcriptors 3 is then recruited to the cytoplasmic region of gp130 and phosphorylated by JAK2 (Kishimoto and Kikutani, 2001). Activated STAT3 forms homodimers, moves into the nucleus and activates transcription of various genes, including the antiapoptotic regulatory protein Bcl-xl *and SOCS-1* (Kishimoto and Kikutani, 2001).

The human *SOCS-1* gene on chromosome 16p13.3 is a single-exon gene encoding 211 amino acids (Endo *et al*, 1997; Naka *et al*, 1997; Starr *et al*, 1997). The gene lies within a large CpG island spanning 2.5 kb. Recently, frequent aberrant methylation of the CpG island of *SOCS-1* gene in hepatocellular carcinoma (HCC) and in multiple myeloma, and it was also demonstrated that the aberrant methylation had growth suppression activity (Yoshikawa *et al*, 2001; Galm *et al*, 2002). These results implicate the JAK/STAT pathway as having growth-promoting properties and suggest that SOCS-1 may normally suppress growth (Bromberg *et al*, 1999; Rottapel *et al*, 2002).

DNA methylation is a major mechanism for the inactivation of tumour-suppressor genes in cancer (Jones and Baylin, 2002). We have previously reported that many benign and malignant pancreatic neoplasms harbour aberrant methylation at multiple genes including *p16*, *preproenkephalin*, *TSLC1*, and others (Fukushima *et al*, 1997; Ueki *et al*, 2000; Ueki *et al*, 2001; Jansen *et al*, 2002). In this study, we determined the prevalence and significance of *SOCS-1* methylation in pancreatic neoplasms.

## MATERIALS AND METHODS

### Cell lines and lymphocytes

Human pancreatic cancer cell lines AsPC1, BxPC3, Capan1, Capan2, CFPAC1, Mia PaCa2 and Panc-1 were obtained from the American Type Culture Collection (Rockville, MD, USA) and Colo357 was purchased from the EACC (Salisbury, UK). Twelve pancreatic carcinoma cell lines (PL1, 3, 4–6, 8–14) were generously provided by Dr Elizabeth Jaffee (Johns Hopkins University). An immortal human pancreatic duct epithelial cell line, HPDE, was kindly provided by Dr Ming-Sound Tsao (University of Toronto, Ontario, Canada). All cell lines, except for HPDE, were cultured in RPMI 1640 medium (Life Technologies, Inc., Gaithersburg, MD, USA) supplemented with 10% fetal bovine serum and antibiotics (100 U/ml penicillin and 100 *μ*g ml^−1^ streptomycin), and incubated at 37°C in a humidified atmosphere of 5% CO_2_ in air. HPDE cells were cultured in keratinocyte serum-free (KSF) medium supplemented by bovine pituitary extract and epidermal growth factor (Gibco-BRL, Grand Island, NY, USA). Lymphocyte DNA was collected from 11 healthy individuals after informed consent as part of an IRB-approved clinical trial that aims to investigate candidate markers of pancreatic cancer.

### Surgically resected pancreatic ductal adenocarcinoma and intraductal papillary mucinous neoplasms

Sixty primary pancreatic adenocarcinomas and 34 intraductal papillary mucinous neoplasms (IPMNs) were obtained from resected surgical specimens at the Johns Hopkins Hospital. The patients with pancreatic ductal adenocarcinoma included 33 men and 27 women; mean age was 65.6 years (range, 38–85 years), and those with IPMN included 17 men and 17 women; mean age was 66.1 years (range, 36–83 years). The IPMNs were histologically divided into three adenomas, 12 borderline neoplasms, five non-invasive and 14 invasive adenocarcinomas according to the WHO classification. Frozen tissues or formalin-fixed paraffin-embedded tissues were microdissected manually by blade and needle to obtain greater than 40% neoplastic cellularity in these primary pancreatic neoplasms. Microdissected tissues were transferred to tube containing 50–100 *μ*l of 1 × TK buffer (200 *μ*g ml^−1^ proteinase K and 0.5% Tween-20), and incubated at 56°C overnight. The tubes were placed in a 100°C block for 10 min for inactivation of the proteinase K.

### Microdissection of normal pancreatic duct epithelia and PanINs

PanINs was classified into four categories of PanIN-1A, -1B, -2 and -3 according to the standardised nomenclature and classification of pancreatic intraepithelial neoplasia (Hruban *et al*, 2001). Serial sections (8 *μ*m) of formalin-fixed and paraffin-embedded tissue were stained with haematoxylin & eosin. Microdissection of normal pancreatic duct epithelium and PanINs including approximately total 200–1500 cells on three slides each was performed with a small blade and needle as previously described and subjected cells to DNA extraction (Fukushima *et al*, 2002). Forty-nine PanINs (eight PanIN-1A, 21 PanIN-1B, 10 PanIN-2 and 10 PanIN-3) were collected from 13 pancreata with ductal adenocarcinoma (mean age of the patients, 64.4 years; range, 50–79 years) and as were 15 samples of normal pancreatic ductal epithelia (mean age of the patients, 60.9 years; range, 36–79 years).

### Methylation-specific PCR

Bisulphite modification of genomic DNA was performed as previously described (Fukushima *et al*, 2002). The bisulphite-treated DNA was amplified with either a methylation-specific or unmethylation-specific primer set at 40 cycles: 95°C for 20 s, 57°C for 30 s and 72°C for 30 s. A final extension step was included for 3 min at 72°C. The methylation-specific primer sequences in exon 1, which lies within the *SOCS-1* CpG island, were 5′-TCGTTCGTACGTCGATTATC-3′ (sense) and 5′-AAAAAAATACCCACGAACTCG-3′ (antisense). The unmethylation-specific primer sequences were 5’-TATTTTGTTTGTATGTTGATTATTG-3’ (sense) and 5’-AAACTCAACACACAACCACTC-3’ (antisense). The unmethylated reaction was predicted to yield a product of 122 bp and the methylated reaction a product of 132 bp. PCR products were resolved in 3% agarose gels, stained with ethidium bromide and visualised under UV illumination. DNA extracted from normal pancreatic tissue treated *in vitro* by *Sss1* methyltransferase (New England Biolabs, Beverly, MA, USA) was used as a positive control for methylated alleles.

### Reverse-transcription–PCR

RNA from pancreatic cancer cell lines was isolated using Trizol Reagent (Life Technologies, Rockville, MD, USA). One microgram of each total RNA was reverse transcribed (RT) using the Superscript II kit (Life Technologies, Rockville, MD, USA). RT–PCR primers for exon 1 were 5′-GACGCCTGCGGATTCTAC-3′ (sense) and 5′-AGCGGCCGGCCTGAAAG-3′ (antisense). A 181 bp was amplified according to the following conditions; an initial denaturation at 95°C for 3 min, then 35 cycles of 95°C for 15 s, 65°C for 30 s and 72°C for 30 s, followed by a final extension step at 72°C for 3 min. PCR mixture contained 5% dimethyl sulphoxide. A glyceraldehyde-3-phosphate dehydrogenase (GAPDH) primer set was run in duplex PCR with the *SOCS-1* primer to provide an internal control for RNA quantity and quality. PCR products were separated in 3% agarose gels.

### 5-aza-2′-deoxycytidine treatment

To confirm that transcriptional repression related to the methylation status of the *SOCS-1* CpG island, *SOCS-1*-methylated cells (PL10 and PL12) were treated with the demethylating agent 5-aza-2′-deoxycytidine (5Aza-dC) at a final concentration of 1.0 *μ*M for 96 h. After treatment, total RNA was extracted from the cells again and compared the *SOCS-1 gene* expression to that of untreated cells.

### Western blotting analysis

Western blotting analysis was performed using antibodies specific for phosphorylated JAK2 (anti-JAK2 pYpY^1007/1008^ rabbit polyclonal antibody, BioSource International, CA, USA) or phosphorylated STAT3 (anti-pStat3, B-7, mouse monoclonal antibody, Santa Cruz Biotechnology, CA, USA) to investigate the JAK2 and STAT3 phosphorylation status after IL-6 treatment (20 ng ml^−1^ recombinant human IL-6 , BD Biosciences, San Diego, CA, USA). After 96 h treatment of IL-6, culture cells (HPDE, Panc1, PL10 and PL12) were lysed in cell lysis buffer (50 *μ*M Tris-HCl, pH 8.0; 50 *μ*M ethylenediamine tetraacetic acid (EDTA); 150 *μ*M NaCl; 50 *μ*l Triton X-100; 5 *μ*l Protease Inhibitor Cocktail) and boiled for 4 min. After 50 *μ*l of each sample was subjected to SDS–polyacrylamide gel electrophoresis, proteins were transferred to nitrocellulose membranes (Amersham Pharmacia Biotech UK Limited, England) and probed with each antibody. After 2 h treatment with the secondary antibody, specific binding was detected by the enhanced chemiluminescence system (SuperSignal®West Pico Luminol/Enhancer Solution and Stable Peroxide Solution, PIERCE, Rockford, IL, USA).

### Examination of cell proliferation in response to exogenous IL-6

To investigate the effect of methylation of *SOCS-1* gene and its silencing on the JAK/STAT pathway, cells were grown with or without adding human IL-6 and the cell proliferation was observed. *SOCS-1*-methylated (PL10 and PL12) or unmethylated (HPDE and Panc1) cells were seeded at equal densities (1 × 10^4^ cells ml^−1^) in six-well plates. After 72 h, cells were incubated in the presence or absence of 20 ng ml^−1^ recombinant human IL-6 (BD Biosciences, San Diego, CA, USA) for 48, 72 or 96 h. To examine the dose-dependent effect on cell proliferation of IL-6, cells were incubated in the existence or absence of 4, 20 or 100 ng ml^−1^ IL-6 for 96 h in the same way. Cells were counted using a haemacytometer (Hausser Scientific, Horsham, PA, USA).

### Statistical analysis

Statistical analysis was performed using the StatView 5.0 statistical software package (SAS Institute Inc., Cary, NC, USA). The unpaired *t-*test was used to determine statistical differences of cell proliferation in between IL-6 treated and untreated cells. A *P*-value of less than 0.05 was considered to indicate statistical significance.

## RESULTS

### Methylation of *SOCS-1* in pancreatic tissues

MSP analysis revealed aberrant methylation of *SOCS-1* in six of 19 (31.6%) pancreatic cancer cell lines (Table 1

**Table 1:** Summary of the results of *SOCS-1* MSP

	***n***	**Methylation of *SOCS-1***	**Frequency**
Pancreatic cancer cell line	19	6	31.6%
Primary pancreatic cancer	60	13	21.7%
IPMNs	34	2^a^	5.9%
PanINs	49	0	0%
Normal pancreatic duct epithelium	15	0	0%
Lymphocytes from healthy people	11	0	0%

**Figure 1:**
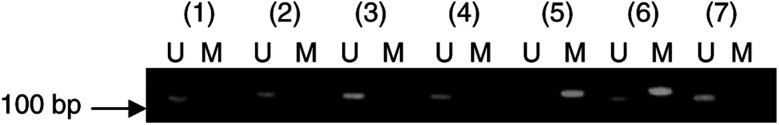
Methylation-specific PCR of *SOCS-1* gene CpG islands. A visible PCR product in lane-U indicates the presence of unmethylated gene promoters; the presence of product in lane-M indicates the presence of promoter methylation. (1) Panc1; (2) BxPC3; (3) Colo357; (4) AsPC1; (5) PL9; (6) PL12; and (7) normal lymphocytes.

IPMNs=intraductal papillary-mucinous neoplasms; PanINs=pancreatic intraepithelial neoplasias.

a IPMN with associated invasive carcinoma.

). The cell lines harbouring methylated *SOCS-1* were PL9, PL10, PL12, Capan1, Capan2 and MiaPaCa2. Complete or almost complete methylation was seen in the cell lines PL9, PL10 and PL12. An example of an MSP analysis is shown in Figure 1

A.

Thirteen of 60 (21.7%) resected primary pancreatic ductal adenocarcinomas showed *SOCS-1* CpG island methylation (Table 1). There was no significant difference in the age of patients with unmethylated *SOCS-1* (65.8 years; range, 38–85 years) and methylated *SOCS-1* (65.1 years; range, 46–83 years) in their primary pancreatic cancers. DNA from normal pancreatic ducts (*n*=16) and from 49 PanINs obtained from the resection specimens of patients with pancreatic adenocarcinoma did not show methylation of CpG island of *SOCS-1* gene (Table 1). Lymphocyte DNA from healthy individuals also only contained unmethylated *SOCS-1* DNA (*n*=11).

Aberrant methylation of the *SOCS-1* CpG islands was detected in two (5.9%) of 34 IPMNs (Table 1). Both of them were IPMNs with associated invasive carcinoma. One of these two patients with methylated *SOCS-1* in their cancer was a patient with Peutz-Jeghers syndrome.

### Relation between aberrant methylation and gene expression of *SOCS-1*

**Figure 2:**
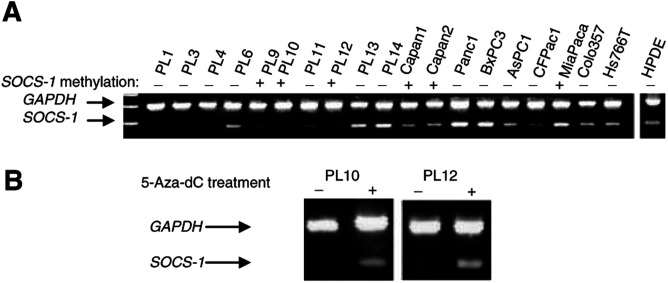
(**A**) *SOCS-1* gene expression in RT–PCR. SOCS-1 gene expression was silenced or strongly reduced in PL1, PL3, PL4, PL9, PL10, PL11 and PL12, and moderately in PL6, Capan1, Capan2, AsPC1 and CFPac1. (**B**) *SOCS-1* gene expression is restored after 5-Aza-dC treatment.

*SOCS-1* gene expression was silenced or markedly reduced in seven of 19 cell lines relative to *GAPDH* by RT–PCR (Figure 1B). Five of the six methylated *SOCS-1* cell lines had markedly reduced or absent *SOCS-1* gene expression. To investigate whether the suppression of *SOCS-1* gene expression was mediated by methylation of CpG islands in human pancreatic cancer cells, we treated the pancreatic cancer cell lines PL10 and PL12 cells with 5-Aza-dC. Both cell lines showed loss of *SOCS-1* gene expression and both harboured methylated *SOCS-1* prior to treatment with 5-Aza-dC. 5-Aza-2′-deoxycytidine restored *SOCS-1* gene expression (Figure 2

). Despite 5-Aza-dC induction of *SOCS-1* gene expression, 5-Aza-dC treatment did not lead to induction of JAK2 or pSTAT3, perhaps because of the relatively modest induction of SOCS-1 gene expression by 5-Aza-dC.

### Effect of exogenously added IL-6 on proliferation of pancreatic cancer cells

**Figure 3:**
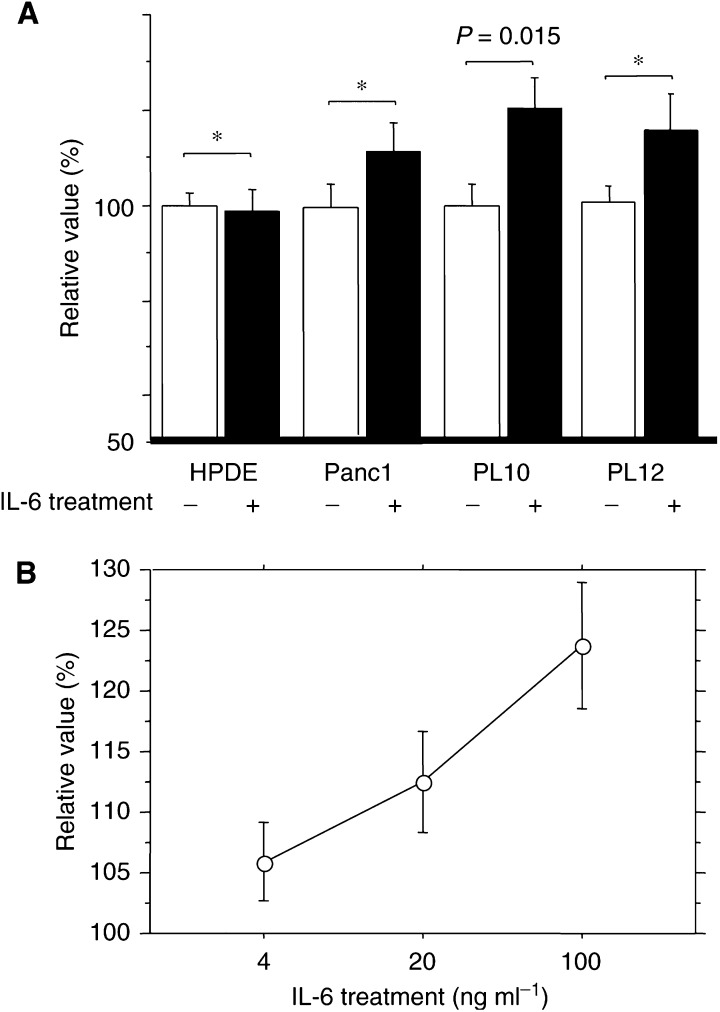
The effect of IL-6 on proliferation of pancreatic cancer cells. (**A**) IL-6 treatment increased PL10 cell number compared to untreated cells (*P*=0.015). Interleukin-6 did not significantly increase the growth of PL12, Panc1 or HPDE. (**B**) Dose-dependent effects of IL-6 on the pancreatic cancer cell line (PL10).

**Figure 4:**
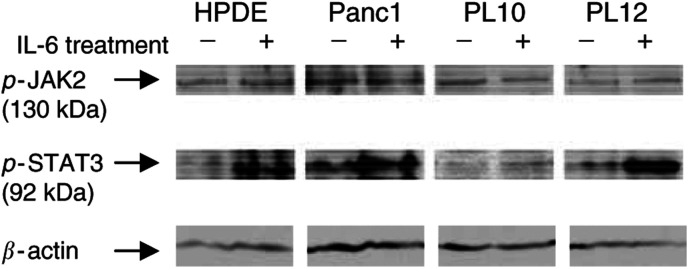
Western blot analysis of phosphorylated JAK2 and STAT3 protein. The 130 kDa band corresponding to phosphorylated JAK2 protein. The 92 kDa band representing phosphorylated STAT3 protein was variably induced after IL-6 treatment.

Treatment of *SOCS-1*-methylated pancreatic cancer cells (PL10 and PL12) and the unmethylated cell line Panc-1 and a non-neoplastic pancreatic ductal epithelial cell line (HPDE) with IL-6 (20 ng ml^−1^) for 48, 72 or 96 h, led to modest but significant increases in PL10 cell growth over control in a time (data not shown) and in a dose-dependent fashion (Figure 3

). After 96 h, IL-6 treatment of PL10 resulted in significant increases in cell number (120.5±21.9%, *P*=0.015) compared to untreated cells, whereas two other pancreatic cancer cell lines, Panc1 and PL12, and the non-neoplastic HPDE cells did not change their growth patterns in response to IL-6 (Panc1: 111±20.8% (*P*=0.146), PL12: 116±7.41% (*P*=0.109), HPDE: 98.7±16.4% (*P*=0.807)) (Figure 3A). Stimulation by IL-6 would be expected to lead to an induction of phosphorylated JAK2 and STAT3. Despite this prediction, Western blotting analysis revealed that phosphorylated JAK2 was only minimally induced after IL-6 treatment in PL12 but not in PL10 (Figure 4

). Phosphorylated STAT3 was present in all cell lines and IL-6 treatment resulted in an increase in pSTAT3 in the SOCS-1-methylated cell lines, PL10 and PL12, but not in the unmethylated cell line Panc-1 (Figure 4). IL-6 treatment also increased pSTAT3 in the pancreatic epithelial non-neoplastic cell line, HPDE (Figure 4).

## DISCUSSION

In this study, we demonstrate that *SOCS-1* is a frequent target for aberrant methylation in pancreatic cancer. We also demonstrate that silencing of *SOCS-1* gene expression occurs in pancreatic cancer cell lines harbouring methylated *SOCS-1* and such silencing could be restored after 5-Aza-dC treatment, indicating that methylation was the likely mechanism by which the *SOCS-1* gene expression was suppressed. Furthermore, we demonstrated that IL-6 induced modest increases in cell proliferation only in pancreatic cancer cells (PL10) harbouring methylated *SOCS-1* gene.

Since SOCS-1 suppresses the JAK/STAT pathway by inhibiting JAK2 activity (Kishimoto and Kikutani, 2001), and since STAT3 is downstream of JAK2 in the JAK/STAT pathway, *SOCS-1* methylation status may affect the level of phosphorylation of STAT3. We observed a moderate induction of phosphorylated STAT3 in PL10 and PL12 after IL-6 treatment with little or no induction in an unmethylated cell line (Panc-1). This is consistent with our hypothesis that pSTAT3 induction by IL-6 would be more likely to occur if SOCS-1 is methylated than if it is unmethylated. We did not observe induction of pSTAT3 or JAK2 by 5-Aza-dC treatment despite induction of SOCS-1, but we suspect that the level of induction of SOCS-1 expression by 5-Aza-dC was insufficient to result in a change in pSTAT3. There was not a clear-cut relation between pSTAT3 levels and SOCS-1 expression and we suspect that this observation reflects the fact that STAT3 is regulated not only by JAK2 but also by other factors including the insulin-like growth factor-1 receptor (IGF-R) and Src (Yu *et al*, 1995).

Methylation of CpG island of *SOCS-1* gene appears to occur late in the development of pancreatic ductal adenocarcinoma. PanINs are preinvasive intraductal lesions (Hruban *et al*, 2001), and PanINs harbour many of the same genetic and epigenetic abnormalities seen in invasive ductal adenocarcinomas such as mutations of the *K-ras* (Moskaluk *et al*, 1997), *p16* (Moskaluk *et al*, 1997; Wilentz *et al*, 1998), *SMAD4* (Wilentz *et al*, 2000), *p53* and *BRCA2* genes (Goggins *et al*, 2000), and aberrant methylation of the *ppENK, p16* and *TSLC1*genes albeit at lower frequencies than those seen in invasive pancreatic cancers (Fukushima *et al*, 2002; Jansen *et al*, 2002). In contrast, in this study we found that PanINs lacked methylation of *SOCS-1* suggesting that if loss of SOCS-1 is a selected event, it does not provide a selective advantage to a pancreatic neoplasm until the neoplasm has evolved into an invasive adenocarcinoma.

IPMN is clinicopathologically distinct neoplasm from ductal adenocarcinoma (Fukushima *et al*, 1997; Longnecker *et al*, 2000). Intraductal papillary mucinous neoplasms show duct ectasia with a papillary growth of neoplastic mucin producing epithelial cells. Patients with IPMNs generally have better prognosis than the patients with usual pancreatic ductal adenocarcinoma; however, some IPMNs evolve into invasive carcinoma and those patients have a poorer outcome. Intraductal papillary mucinous neoplasms have been shown to have similar methylation patterns to PanINs and usual pancreatic adenocarcinomas. For example, *ppENK* and *p16* methylation occurs at a higher frequency in high-grade IPMNs (carcinomas) than it does in low-grade IPMNs (adenoma or borderline neoplasms) (Sato *et al*, 2002). In this study, we detected aberrant methylation of *SOCS-1* gene in only two of 14 IPMNs that had an associated infiltrating carcinoma. None of the 20 benign or borderline IPMNs without an associated invasive carcinoma harboured *SOCS-1* methylation. This suggests that, just as is true for usual ductal pancreatic adenocarcinoma, aberrant methylation of *SOCS-1* gene may occur late in the development of IPMNs.

Suppressor of cytokine signalling-1 gene can be added to the increasing list of genes that are aberrantly methylated in pancreatic adenocarcinomas. This list includes *ppENK, p16, hMLH1, RAR beta* (Ueki *et al*, 2000, 2001)*, cyclin D2* (Matsubayashi *et al*, 2003) and *TSLC1* (Jansen *et al*, 2002). In addition to understanding the biological significance of aberrant methylation in pancreatic adenocarcinoma, the detection of aberrantly methylated DNA in secondary fluids such as the pancreatic juice of patients with pancreatic disease may aid in the early diagnosis of pancreatic cancer (Fukushima *et al*, 2003). Since *SOCS-1* methylation is only found in a subset of pancreatic adenocarcinomas, its utility as a diagnostic marker would only be as part of a panel of other aberrantly methylated genes that are specifically methylated in pancreatic cancer but not in normal pancreas.

In conclusion, *SOCS-1* is commonly methylated in pancreatic adenocarcinoma and loss of SOCS-1 in pancreatic cancer is moderately associated with increased IL-6-mediated growth. Since *SOCS-1* methylation becomes manifest only at the invasive carcinoma stage, detection of methylated *SOCS-1* may aid in the early detection of invasive pancreatic adenocarcinoma.
